# Measuring Surface Area of Skin Lesions with 2D and 3D Algorithms

**DOI:** 10.1155/2019/4035148

**Published:** 2019-01-15

**Authors:** Houman Mirzaalian Dastjerdi, Dominique Töpfer, Stefan J. Rupitsch, Andreas Maier

**Affiliations:** ^1^Friedrich-Alexander-Universität Erlangen-Nürnberg (FAU), Lehrstuhl für Informatik 5 (Mustererkennung), Germany; ^2^Softgate gmbh, Erlangen, Germany; ^3^Friedrich-Alexander-Universität Erlangen-Nürnberg (FAU), Lehrstuhl für Sensorik (LSE), Germany

## Abstract

**Purpose:**

The treatment of skin lesions of various kinds is a common task in clinical routine. Apart from wound care, the assessment of treatment efficacy plays an important role. In this paper, we present a new approach to measure the skin lesion surface in two and three dimensions.

**Methods:**

For the 2D approach, a single photo containing a flexible paper ruler is taken. After semi-automatic segmentation of the lesion, evaluation is based on local scale estimation using the ruler. For the 3D approach, reconstruction is based on Structure from Motion. Roughly outlining the region of interest around the lesion is required for both methods.

**Results:**

The measurement evaluation was performed on 117 phantom images and five phantom videos for 2D and 3D approach, respectively. We found an absolute error of 0.99±1.18  cm^2^ and a relative error 9.89± 9.31% for 2D. These errors are <1  cm^2^ and <5% for five test phantoms in our 3D case. As expected, the error of 2D surface area measurement increased by approximately 10% for wounds on the bent surface compared to wounds on the flat surface. Using our method, the only user interaction is to roughly outline the region of interest around the lesion.

**Conclusions:**

We developed a new wound segmentation and surface area measurement technique for skin lesions even on a bent surface. The 2D technique provides the user with a fast, user-friendly segmentation and measurement tool with reasonable accuracy for home care assessment of treatment. For 3D only preliminary results could be provided. Measurements were only based on phantoms and have to be repeated with real clinical data.

## 1. Introduction

Clinicians deal with several kinds of lesions such as diabetes, pressure ulcer and trauma wound, etc. Most of these lesions can be considered chronic wounds and therefore, a periodic monitoring and wound assessment play an important role in performing a diagnosis and reevaluate the treatment strategy. It also may enhance the quality of patient care providing more objective assessments of treatments [1].

Outlining the lesions depends on clinician's opinion and may vary among different operators or even for the same operator when outlining the same lesion multiple times. Wannous et al. [2] reported the overlap score of 70% between different expert users. A more precise monitoring treatment may be obtained using an automatic wound segmentation technique which is a critical part in order to achieve a reproducible result.

The simplest and cheapest method of surface area measurement is manually calculating the linear wound dimensions (i.e., length and width) with a ruler [3]. However, this method is time-consuming and its accuracy and reliability can vary according to the subjective determination of the wound edges. Therefore, and because of the decreasing price of digital cameras, photographic techniques gained more and more attention for wound surface measurements [4]. Foltynski et al. [5] placed a transparent double-layer grid film over the wound. The wound outline on the film is then traced manually. Next, a picture is taken from the flat grid film including the wound contour. Finally, the surface area is calculated by processing the picture using a graphics software AreaMe. The mean relative error is calculated for area measurement of 108 samples with -3.4% for the AreaMe method based on only phantom images. It is also time-consuming and due to direct contact of the film with the lesion, it may hurt the patient. Foltynski et al. [6] proposed a new method of wound measurement using two parallel rulers below and above the phantom wound images to improve the precision of area measurement approximately four times compared to the measurement based on one ruler for calibration. However, this result is reported for a flat skin. For curved areas, they report their method to be outperformed by others.

A noncontact wound measurement method is performed using a laser scanner system named FastSCAN Polhemus [7]. This allows for obtaining a precise 3D model from which the area can be computed. Although this method has developed as a fast, accurate, and noncontact way of wound measurement and documentation, it requires a specific and expensive equipment which becomes unhandy, for example, in home care applications.

Wannous et al. [2] presented a fully automatic wound segmentation using Support Vector Machine (SVM) based on a single image. The best achieved result of SVM wound segmentation is obtained with 77%, 92%, and 88% for sensitivity, specificity, and overall accuracy, respectively. In [8], which is a stereophotogrammetric technique, a 3D assessment of skin wounds is proposed using a standard digital camera. However, this method entails the development of a robust image processing chain including the use of color correction to improve the results. For the 3D surface evaluation, a precision error of 3% was obtained.

In [9], Sirazitdinova et al. present a system for 3D wound imaging using low-cost mobile devices based on dense reconstruction in real time. They apply color correction [10, 11] for easier segmentation. This also allows for the separation of different tissues in the wound (granulation, necrosis, and slough). So far, only global thresholding has been applied which may later be replaced by more precise algorithms.

There are also several commercial applications for wound assessment using a single photo such as “imito” (http://imito.io) and “LesionMeter” (http://lesionmeter.com) based on a specific scale descriptor (e.g., QR code). However, both methods require manual delineation of the wound and it is unlikely that other than perspective correction is performed. Also, to the best of our knowledge, so far no result on precision or accuracy was published. Nixon et al. [12] evaluated another commercial device, the Silhouette®. This device uses a combination of laser and photo cameras in order to measure the wound surface area. Percentage error is reported less than 5% for the areas on different curvature skin; however, lesion segmentation has to be performed manually.

Considering all above-mentioned limitations of existing methods, this work aims to provide an easy, convenient, fast, and low-cost approach for semi-automatic wound segmentation and area measurement with high accuracy based on a single image. As the details of the segmentation method have already been described in [13], only a brief presentation of the method's design will be given here, to focus particularly on the details of surface area measurement algorithms. The input image contains a flexible paper ruler to estimate the area of a lesion on a curved surface. This paper proposes a novel checkerboard detection algorithm that is independent of the number of visible squares in the ruler. Note that, from only one image, one cannot easily obtain depth information which increases a measurement error, especially in the case of a curved surface wound. To address to this problem, a second approach is implemented in 3D to improve the accuracy, especially in case of having nonplanar (bent) surfaces. However, time pressure is usually a problem in clinical routine and both manual delineation and taking multiple images from different angles can become time-consuming when it has to be done for many patients. In experiments, the accuracy of this method is evaluated for wound segmentation and area measurement [13]. Zenteno et al. [14] have compared VisualSFM and the laser scanner approach. They showed VisualSFM and laser scanner have the comparable result.

## 2. Materials and Methods

Our new 2D approach works based on only a single image taken with a commercial handheld digital camera or smartphone. For helping to estimate the local scale of the image even for curved surfaces, a flexible paper ruler is used. To keep measurement errors such as lightning effects, perspective distortion for bent wound, etc. as low as possible, some conditions must be met during the image acquisition: (1)The camera shall be perpendicular to the wound surface. (2)The wound shall be located in the center of the image (for minimizing lens distortion). (3)The ruler has to be placed parallel to the largest wound diameter as close as possible to it. Ideally, it should also reflect the curvature of the surface.

The proposed 3D method in this study is based on a video taken from a lesion. Instead of the flexible ruler, only a reference of known size has to be contained. We were provided with five videos of wound lesion from clinical routine. The camera has to move in order to get images of the lesion from different angles.

Both, the 2D and the 3D approach have been developed based on real clinical data. However, as we only had access to very few images containing the ruler and videos, we focused on evaluation based on phantom images.

### 2.1. Lesion Segmentation Method

**Figure 1:**
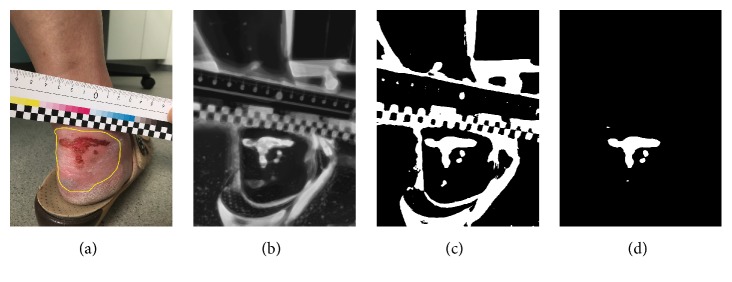
(a) Original image. (b) Probability map (result of Random Forest classifier). (c) The result of Otsu's filter. (d) Segmentation result after applying the ROI.

The segmentation method was already described in [13] and as here no new results are reported, it shall only be explained shortly for completeness. It is based on Random Forest (RF) classification [15]. The RF is trained such that it classifies images into only two groups, wound and skin [13]. Therefore, the output of the RF for background or ruler pixels is not meaningful and they are discarded using a Region of Interest (ROI) defined by the user by roughly drawing a contour around the lesion; see Figure 1(a).

The training data used in RF is generated based on a semi-automatic segmentation algorithm. In order to facilitate generating the ground truth mask, the modified version of the RW algorithm using Quaternion Color Curvature (QCC) [16] has been used during the wound segmentation process (training). For training the RF, the feature vectors are generated by applying a filter bank on both RGB and LAB color space as some features are more prominent in LAB image rather than RGB images due to the variety of wounds. The output of the RF (Figure 1(b)) is a probability map defining how likely it is that a single pixel belongs to wound or skin. Otsu's thresholding method is then applied to extract the binary mask from the probability map (Figure 1(c)). Finally, by applying the ROI (as a mask) to the output of Otsu's thresholding, wound area is determined; see Figure 1(d).

### 2.2. Measurement Method

#### 2.2.1. 2D Surface Area Measurement

**Figure 2:**
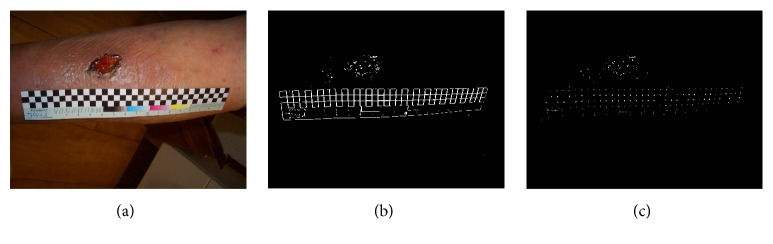
(a) Original wound image and the ruler. (b) Result of structure tensor filter for one high eigenvalue. (c) The result of structure tensor filter for two high eigenvalues.

The flexible paper ruler is used to obtain the local scale of the wound. For easy detection, the ruler contains a checkboard pattern with known square size, Figure 2(a). For extracting the ruler, the Structure Tensor filter [17] is applied to the grayscale image. The orientation information of edges and corners is obtained from Structure Tensor filter output. In the next step, the eigenvalues of the Structure Tensor output are computed which gives the ruler skeleton (if only one eigenvalue is large) shown in Figure 2(b) and the corner points (if both eigenvalues are large) Figure 2(c).

**Figure 3:**
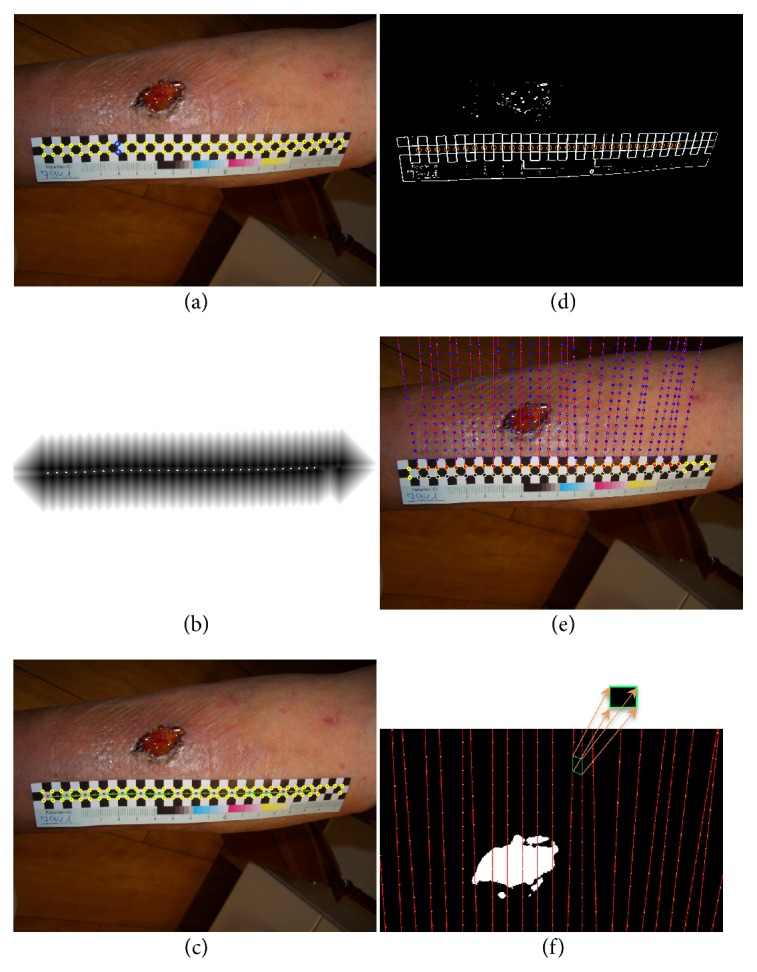
(a) Identified checkboard points and two corresponding corner points *p*_1_ and *p*_2_ shown with blue circles. (b) The result of applying DT filter on the corner points. The white dots show the computed local maxima. (c) The fitted curve to the local maxima of the distance transform point result (cline). (d) The red circle windows are applied at the location of each detected intensity change in the high eigenvalue image. (e) Creating a grid pattern by extrapolating each pair of corresponding points over the wound. (f) Each quadrilateral in the grid is unwarped to a square with known size.

To specify the corner points which belong to the checkboard, a square window with a fixed size is placed at the location of each detected corner point. Then, the mean intensities of pixels located on the window corner are compared diagonally and based on this comparison, checkboard points are identified; see Figure 3(a).

Considering that our checkboard has three rows, the corner points are aligned in two rows. In order to identify pairs of corresponding points (*p*_1_, *p*_2_) with *p*_1_ lying on the upper and *p*_2_ lying on the lower row, the Distance Transform (DT) is applied to the corner points. The local maxima of DT are then calculated which gives a set of points that lie on a line between upper and lower corner points; see Figure 3(b). Using spline interpolation, a curve is fitted to these obtained local maxima points, Figure 3(c). Moving along this curve on the wound image allows finding pairs of corresponding points by detecting intensity changes across checkerboard edges.

Having the high eigenvalue image of the structure tensor filter, a circle window is applied at the location of each detected intensity change which gives the actual edges on the ruler checkboard, Figure 3(d). The window size is selected according to the smallest distance between corner points.

We used Principal Component Analysis (PCA) to obtain a line along the checkerboard edges. Then by moving along both positive and negative directions of the normal vector, the pairs of corresponding points are determined.

Checkboard points provide the scale information in two directions x and y. Using a heuristic approach, the local measurement parameters are extrapolated along the line defined by *p*_1_ and *p*_2_, by placing *p*_3_, *p*_4_,… equidistantly using *d* = ‖*p*_1_ − *p*_2_‖_2_; see Figure 3(e).

For measuring the wound area, each quadrilateral in the grid is unwarped from perspective distortion [18] and mapped to a square; see Figure 3(f). As the “true” size of the square is known from the definition of the ruler, measuring comes down to counting the wound pixels covered by the square and using the formula:

(1) $$\begin{array}{c} A_{w}=\frac{N_{w}}{N}\cdot area\;\;of\;\;square, \end{array}$$

where *N* and *N*_*w*_ are the total number of pixels and wound pixels inside each square of the checkerboard, respectively. *A*_*w*_ is the surface area of a wound inside each quadrilateral in the grid after getting unwarped. Adding these results for all squares yields the surface area of the lesion.

#### 2.2.2. 3D Surface Area Measurement

**Figure 4:**
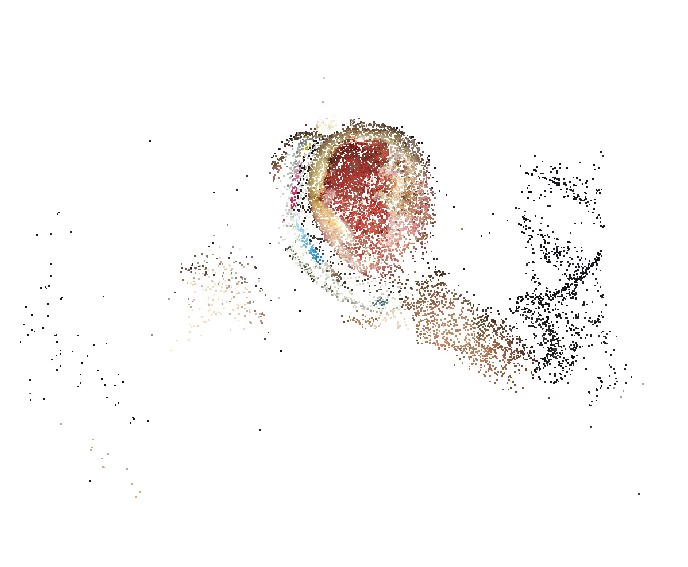
The obtained 3D point cloud from the SfM algorithm.

The proposed 3D reconstruction is based on a technique named Structure from Motion (SfM) which creates a 3D point cloud from multiple images of a scene taken from different angles [19]. The first step is to extract a sequence of images from the video. In this work, the point cloud reconstruction is performed using the SIFT algorithm for feature extraction and RANSAC for feature matching. The obtained point cloud (Figure 4) may have some isolated regions. Those points which have few neighbors are eliminated by discarding points with too few neighbors in a specified neighborhood. For better specification of the wound area, the point cloud is colorized according to the color information of corresponding pixels in the image sequence. Basically, each 3D point's color is determined by calculating the mean of the colors from the corresponding pixels in the images. This stage is called color reconstruction.

**Figure 5:**
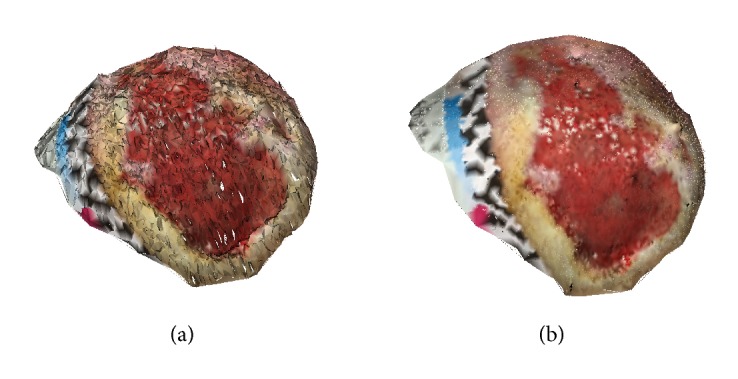
(a) The result of Delaunay triangulation algorithm which is a rough surface including some holes. (b) The smooth and integrated surface after filtering.

In the next step, a triangle mesh is generated from the remaining points by applying the Delaunay algorithm [20]. The reconstructed surface may be rough and may contain holes; see Figure 5(a). To remove the holes and get an integrated surface, a Laplacian smoothing filter is used as well as hole filling (Figure 5(b)).

**Figure 6:**
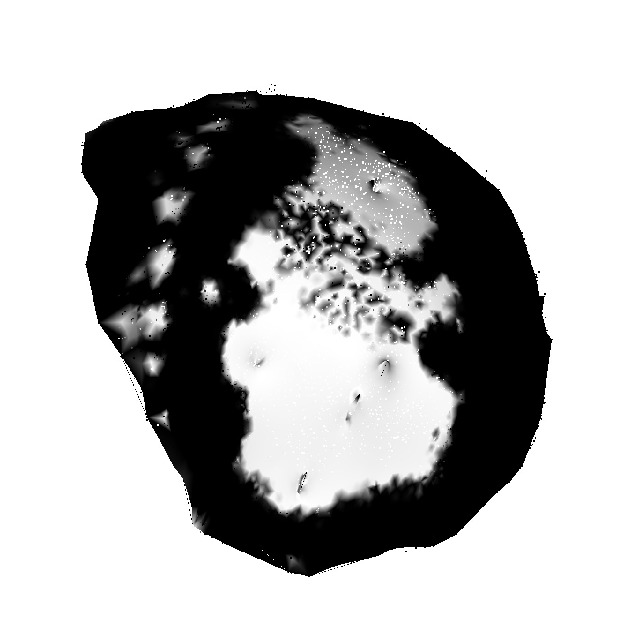
The grayscale 3D surface after color reconstruction based on binary segmentation mask sequence.

In order to do the segmentation in 3D, the first step is to apply the RF that is used in the 2D approach, to all images of the sequence which gives the binary mask for all the images. The color reconstruction is repeated according to the binary mask sequence which results in a grayscale surface; see Figure 6.

As the same method as for the 2D segmentation is applied here, it is also necessary to have the ROI in order to discard the irrelevant areas. For now, outlining was done in all images in the sequence manually. Providing a more user-friendly way (for example, directly on the triangle mesh or on a flattened map) was out of the scope of this work. The surface area can now be obtained as the sum of areas of individual areas of triangles having at least two white (gray value > 127) vertices.

### 2.3. Phantom Creation

**Figure 7:**
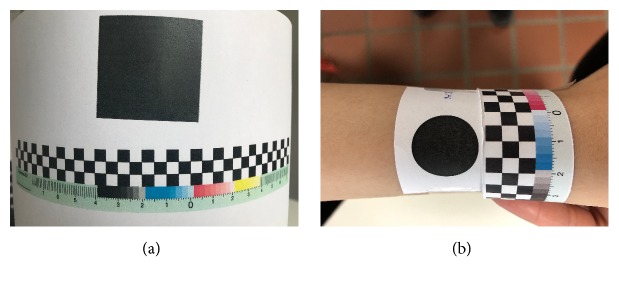
(a) A phantom example. (b) A phantom image which is placed on a person's arm.

For our 2D measurement approach, a set of phantom images were used as the true size of the lesions in clinical images is usually not known. These phantoms contain geometric shapes (i.e. ellipse, rectangular, etc.) of known size for performing evaluation of the proposed algorithm. Different phantom images with distinct curvature (the low and high curvatures are cylinders with radius of approximately 19.5 and 8.5 cm, respectively) were taken for testing our measurement approach.  Figure 7(a) shows an example of a phantom which has a low curvature surface. Segmentation was done by simple thresholding. For each image, a proper threshold was chosen independently by a human operator. For the evaluation of reproducibility, a different set of three phantoms were placed on a person's arm (Figure 7(b)) and five random users were asked to take photos after reading the acquisition instruction. A preliminary evaluation of the 3D approach was based on five phantom images. For the algorithm to work, the area has to have trackable features, which is why small structures were included in the area. Also here, segmentation was done by thresholding, choosing a suitable threshold for each dataset manually.

## 3. Results

### 3.1. 2D Area Measurement Validation

**Figure 8:**
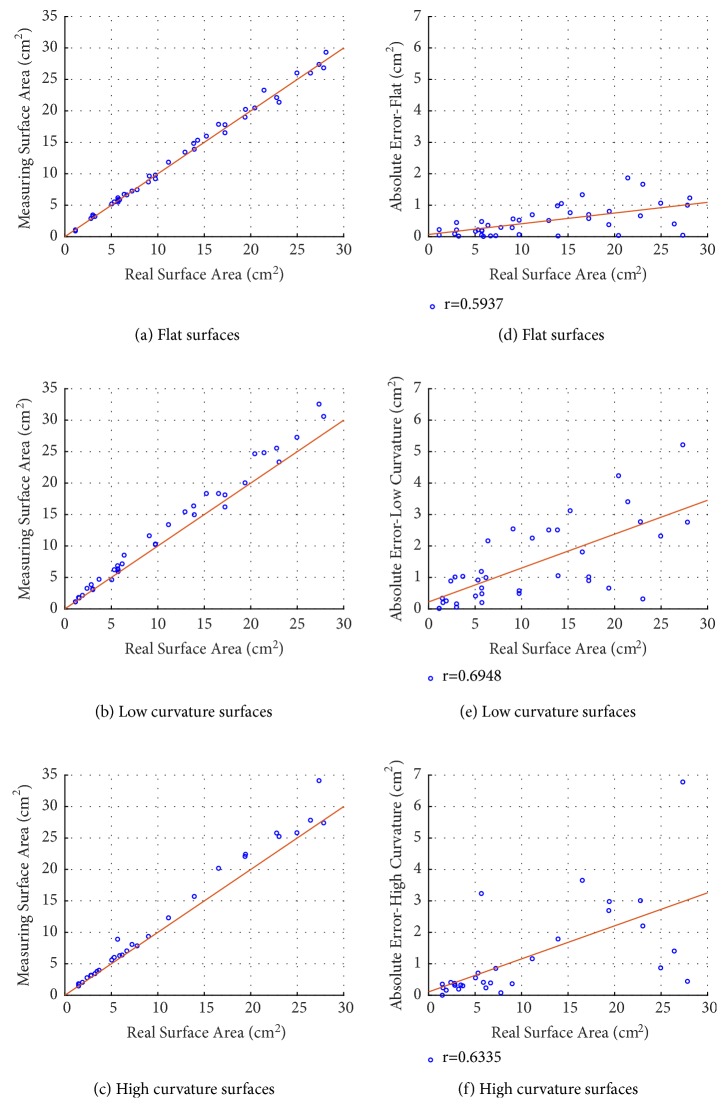
Comparison between the real surface area and the measured surface area for flat (a), low curvature (b), and high curvature (c) surfaces (lines represent identities). Computed absolute error for the real surface area and the measuring surface area for flat (d), low curvature (e), and high curvature (f) surfaces (lines represent regression lines).

The 2D area measurement evaluation was performed on 117 phantom images with different geometric shapes of different sizes (ranging within 1.13 − 28.09  cm^2^). From these 117 images, 8 images were excluded due to converging grid pattern in the phantom area or phantom area being outside of the grid. These phantom images were taken with an iPhone7's and iPhoneX's cameras in order to simulate use of different cameras in practice. For simulating lesions on curved surfaces, and also for simulating practical use, the angle of the camera was varied slightly. For the measurement validation (e.g., for flat, low, and high curvature) an absolute error of 0.99 ± 1.18  cm^2^ and a relative error of 9.86 ± 9.31% were obtained.  Table 1 shows the results grouped into flat images and images with lower and higher curvature.  Figure 8 shows the comparison between the size and the absolute error of real area and the measured area, respectively.

To evaluate the reproducibility in our 2D measurement approach, three new phantoms are placed on different parts of one's hand having different curvature. Then five random users (no clinicians or nurses) were asked to acquire images independently based on the image acquisition conditions mentioned in Section 2.  Table 2 shows the result of 2D measurement for images taken by different users. For three images, one ruler point was not detected correctly due to reflection. This was corrected manually by darkening the image locally.

### 3.2. 3D Area Measurement Validation

Our validation in 3D was performed based on five phantoms of different curvature and size (ranging within 10.8 − 21.6  cm^2^). We found the mean of 0.63 ± 0.16  cm^2^ and 4.22 ± 0.45% for an absolute error a mean relative error, respectively. The relative errors and absolute errors are shown in Table 3.

## 4. Discussion and Conclusion

The main subject of this work was to investigate a new method of wound surface area measurement in 2D and 3D. The idea of 2D measurement is based on the estimation of the surface area using a flexible paper ruler placed close to the lesion. We proposed a fast, convenient, and low-cost tool which can be also used for home care applications with an acceptable error. Apart from the area obtained from the segmentation scheme, our 2D measurement result depends on ruler detection and extrapolation. In comparison to available checkboard determination methods, our ruler detection approach has one main advantage; our measurement technique is independent of the number of checkboard squares visible in a wound image especially in case of bent wounds.

Through our 2D study, a different approach for extrapolating the ruler points based on the cross ratio was studied [21]; as this approach was quickly found to be less stable and to have a greater error, it was not pursued any further.

In this paper, we have only addressed evaluation based on phantom images as for real clinical data the true size of the lesions could not be obtained. In order to still simulate a realistic setting, during the phantom image acquisition, the distance of the camera to the wound was not controlled. Palmer et al. [22] illustrated the influence of image acquisition on the accuracy of estimation which can increase the error of measurements more than 10%.

We did not have access to a sufficiently large set of images of one lesion photographed by different persons, and reproducibility could not be performed on clinical images. However, reproducibility is evaluated in Table 2 for phantom images. This, of course, leads to an oversimplification of the segmentation process which is why we concentrated on evaluation of the measurement in this paper.

For measuring the area, this algorithm is still lacking the possibility to reject images not taken perpendicular to the wound surface. If the angle is only slightly changed, the errors can quickly increase especially for large lesions or highly curved surfaces. Foltynski [23] showed how to decrease the error of camera tilt angle with the help of the calibration coefficient; however, this was out of the scope of this study. Further research with real clinical data is necessary for validating the 2D measurement method.

The 3D approach was implemented based on only five wound videos. The true size of the lesion was unknown. Therefore, we evaluated our 3D area measurement approach using phantom videos. As expected, the error was lower than for the 2D approach. It should be mentioned that there is still room for improvement, as the segmentation was performed just with a global threshold for the whole image sequence and the phantom setup had to be adjusted to the way the algorithm works. This led to slightly wrong segmentation results in the border of the phantom area. It is likely that segmentation works better for real videos. However it should be noted that the 3D segmentation is obtained from the 2D segmentation of each frame. This raises the question of the influence of bad segmentations on the final result. Due to the lack of clinical data, this question could not be addressed and needs further attention in future research. Clearly, the evaluation has to be performed on a larger dataset and also on real clinical data with multiple videos of the lesions (also over time).

Moreover, in this method, no full automatic processing pipeline has been implemented so far and, for example, the ROI was drawn manually in all images of a sequence. Therefore, as further research for the 3D approach, it is first necessary to simplify outlining the ROI. This could be done, for example, by obtaining a large image from the sequence using image stitching or by providing tools for outlining on the 3D mesh directly. The reference length used for obtaining the scale factor has been measured manually (using Meshlab) and it should be found automatically in the final approach.

Since Zenteno et al. [14] have shown that 3D measurement result has a quality close to laser scanner techniques. Due to limitations we had at this stage, i.e., having access to the laser-scanner images, relying on [14], we hypothesized that our 3D measurements provide an estimation of the laser scanner measurements. This hypothesis helps us to have an indirect comparison between our results and the results by the laser scanner. Also, [2, 8] demonstrated that the measurement error is approximately 10% for available photographic techniques and the precision may vary with wound size. Due to the lack of evaluation on real clinical data for our method, comparison between our results and theirs is not possible.

For the final application, the user should also be able to correct wrong or missing parts of the segmentation manually for both, the 2D and 3D approach. Implementation of such tools was out of the scope of this paper. Last but not least, this paper did not focus on the runtime optimization of applications. For 2D segmentation and measurement, this algorithm works for approximately 25 seconds and for the 3D approach for 5 minutes for approximately 110 images running on a MacBook Pro computer (CPU 3.1 GHz). It is expected that the final application can estimate the area of the wound faster than manual annotation.

## Figures and Tables

**Table 1:** Results for ruler-based measurements. Values for absolute and relative error are given as mean ± standard deviation. Exclusion is due to converging the grid pattern in the phantom area or phantom area being outside of the grid.

Type of image	Absolute error	Relative error	Min Area	Max Area
	(cm^2^)	%	(cm^2^)	(cm^2^)
Flat (N=41)	0.49±0.47	4.48±3.90	1.13	28.09
Low Curvature (N=40, 3 excl.)	1.39±1.28	14.02±9.91	1.13	27.84
High Curvature (N=36, 5 excl.)	1.19±1.49	12.00±10.42	1.47	27.84

All (N=109)	0.99±1.18	9.86±9.31	1.13	28.09

**Table 2:** Image acquisition by random users (not clinicians) in order to evaluate the reproducibility. For three images (bold numbers), one ruler point was not detected correctly due to reflection (manually corrected).

User	Phantom 1	Phantom 2	Phantom 3
	3.46 (cm^2^)	3.68 (cm^2^)	6.61 (cm^2^)
1	2.90	**3.80**	7.76
2	3.68	4.04	7.04
3	3.66	3.53	**6.25**
4	3.28	3.52	**6.16**
5	3.45	3.52	6.46

**Table 3:** Area measurement results from five phantom videos.

Phantom	Curvature	Real Size	Measured Size	Absolute error	Relative error
		(cm^2^)	(cm^2^)	(cm^2^)	%
1	Low	10.8	11.29	0.49	4.54
2	Low	16.2	15.58	0.62	3.82
3	Low	21.6	20.70	0.90	4.16
4	High	10.8	11.32	0.52	4.81
5	High	16.2	15.59	0.61	3.77

## Data Availability

The software and data used in this study are not available as they were created as part of a paid customer project of softgate gmbh.
